# Essential not Supplemental: Medicare Advantage Members’ Use of Non-Emergency Medical Transportation (NEMT)

**DOI:** 10.1007/s11606-023-08321-1

**Published:** 2023-07-18

**Authors:** Na’amah Razon, Laura M. Gottlieb, Taressa Fraze

**Affiliations:** 1 Department of Family and Community Medicine, University of California, Davis, Davis, CA USA; 2 Department of Family and Community Medicine, University of California, San Francisco, San Francisco, CA USA; 3 Social Interventions Research and Evaluation Network (SIREN), San Francisco, CA USA; 4 Philip R. Lee Institute for Health Policy Studies, San Francisco, CA USA

**Keywords:** transportation, social risk, medicare advantage, supplemental benefits

## Abstract

**Background:**

Over five million people in the USA miss or delay medical care because of a lack of transportation. Transportation barriers are especially relevant to Medicare Advantage (MA) health plan enrollees, who are more likely to live with multiple chronic conditions and experience mobility challenges. Non-Emergency Medical Transportation (NEMT) helps to address transportation gaps by providing rides to and from routine medical care (for example, medical appointments, laboratory tests, and pharmacy visits) and has been added as a supplemental benefit to some MA health plans.

**Objective:**

We aimed to characterize MA enrollees’ experiences with supplemental NEMT benefits.

**Design:**

Qualitative interviews focused on participants’ experiences with existing NEMT benefits, transportation, and mobility.

**Participants:**

Twenty-one MA enrollees who used their MA NEMT benefit in 2019 and who remained eligible for ongoing transportation benefits through 2021.

**Approach:**

Using purposive sampling from a list of eligible participants, we recruited individuals who used their MA NEMT benefit in 2019 and who remained eligible for benefit-covered transportation services through 2021.

**Key Results:**

Participants considered NEMT an essential service, particularly because these services helped them decrease social isolation, reduce financial insecurity, and manage their own medical needs. Navigating logistical challenges associated with arranging NEMT services required participants to commit considerable time and energy and limited the effectiveness and reliability of NEMT.

**Conclusion:**

Participants described NEMT as a valued service essential to their ability to access health care. They suggested ways to increase service flexibility and reliability that could inform future NEMT policy and practice. As health systems and payers learn how to best address social risks, particularly as the US population ages, our findings underscore the importance of NEMT services and highlight opportunities to advance comprehensive transportation solutions for MA participants.

## INTRODUCTION

Transportation is a foundational resource that connects individuals to their communities. It impacts education, employment, food, isolation, and housing opportunities and is a powerful influencer on access to health care services. Indeed, more than five million people in the USA miss or delay medical care because they lack transportation.^1^ As a result, individuals experiencing transportation insecurity have higher rates of potentially avoidable hospitalizations and emergency department (ED) visits.^2^

Policymakers and payers have long been interested in finding ways to address transportation barriers as one component of multi-pronged strategies to improve health care access and outcomes. As early as 1966, federal mandates required state Medicaid agencies to provide subsidized rides to members through non-emergency medical transportation (NEMT) benefits. NEMT helps to address transportation gaps by providing rides to and from routine medical care (for example, to primary care appointments, laboratory tests, and pharmacy visits).^3,4^ Each state Medicaid program can implement NEMT either by providing direct rides, contracting with a mobility broker, or by collaborating with local transit agencies. While Medicare has historically not provided NEMT services to its beneficiaries, through supplemental benefits, Medicare Advantage (MA) plans can offer NEMT to mitigate the health impact of transportation barriers.^5^ Since MA enrollees are typically seniors with multiple chronic conditions and decreased mobility, MA enrollees are particularly reliant on transportation support to access health care services. Approximately half of all MA plans offered NEMT in 2022.^6^

Prior research indicates that transportation is a barrier to equitable health access.^7,8^ One recent study focused on an MA population found that of all health-related social needs (HRSNs; defined as individual-level, unmet adverse social needs) transportation insecurity was the need most closely associated with increased ED visits and avoidable hospital stays.^2^ Another study found that transportation and housing are the two social risks least likely to change and therefore might be especially impactful intervention targets.^9^ The growing body of evidence demonstrating the links between social risk and health outcomes has prompted multiple national standard-setting organizations to propose quality measures related to social risks, including measures that include transportation screening and interventions. For example, transportation is one of three core social risk domains included in the recent Healthcare Effectiveness Data and Information Set (HEDIS) program for health plans and quality measures in CMS value-based and Medicare Advantage programs.^10^ Despite the potential impact of transportation interventions, two recent reviews highlight major evidence gaps in the design and delivery of transportation interventions.^11,12^

In considering how to best address transportation insecurity, it is important to highlight the distinction between social risk and social need.^13^ The first, like HRSN, is an individual adverse social determinant of health, often identified through a social risk screening tool. The latter depends on the preference of an individual in addressing social risks. The healthcare system currently lacks a robust understanding of the range of transportation insecurity patients face, their experiences navigating MA NEMT, and what they see as successful transportation support. We collaborated with a national healthcare payer that offers supplemental NEMT benefits via multiple MA plans to better understand members’ transportation needs and experiences with NEMT in an effort to fill these knowledge gaps.

## METHODS

### Recruitment

Using purposive sampling from a list of 200 eligible participants provided by the MA plan, we recruited individuals who used their MA NEMT benefit in 2019 and who remained eligible for ongoing transportation benefits through 2021. To participate in the study, individuals needed to have used their NEMT benefits at least once in 2019; many used, or attempted to use, the benefit multiple times since they remained eligible for services the entire study period (2019–2021). We recruited patients who used NEMT services in 2019 because it was the year prior to the COVID-19 pandemic which impacted patients’ pattern of health care utilization in response to the global emergency. We aimed to have representation in population density, race, sex, income, and medical complexity (defined as a number of chronic conditions and hospitalizations in the past year). The MA payer provided these demographic data. The study team also asked each interviewee to self-identify race, ethnicity, and income to ensure accurate and nuanced information. We used this information to make decisions about how to target recruitment to capture a diversity of perspectives (race and ethnicity, urban/rural, income level). All individuals received NEMT service through a large NEMT broker in the USA. A third-party vendor outreached to eligible members and scheduled interviews. Two individuals on the research team, a PhD-level anthropologist and a PhD-level health services researcher, conducted all interviews by phone between April and May 2021. Individuals could decline participation during the initial outreach effort or at any point during the study recruitment or interview process. All interviews were audio recorded with participants’ verbal consent.

Drawing on prior literature,^8,14^ the interview guide domains explored multiple dimensions of transportation services that individuals used to access healthcare and non-healthcare services. We included healthcare and non-healthcare transportation needs to understand comprehensively how people address their transportation needs and to capture the broader health domains such as food access and social support that NEMT does not permit. Typically, NEMT is only provided to/from healthcare facilities or to/from health-related services like laboratory or pharmacy. Interviews lasted between twenty minutes and one hour. We adapted the interview guide after the first five interviews to improve clarity and flow. We continued to interview individuals until thematic saturation was achieved by the emergence of consistent themes without new domains. We compensated participants with a $50 gift certificate to a retail vendor.

### Data Analysis

A HIPAA-compliant service transcribed all interviews. The research team reviewed all transcribed interviews to ensure all patient-identifying information was removed. The interviewers met regularly throughout the interview process to discuss the interview guide and emerging themes. Each interviewer completed a memo summarizing key themes immediately after each interview. Memos initially began as unstructured to highlight key interview domains and with time became more structured as common themes emerged during the interviews. Drawing on these memos and existing literature, we used an inductive approach to develop an initial codebook focused on key domains and informed by a transportation insecurity framework.^8,14^ Using Atlas.ti software, two team members coded the transcribed interviews. Initially, two team members coded the same two interviews individually and then met to compare the application of the codebook and address differences. Once we reached a consensus on the application of the coding schema for these two interviews, each coder received three additional interviews for review. We then met to discuss the coding process for these six interviews to ensure consistent application of the codebook. The remaining thirteen transcripts were then divided between the two team members (and read by only one team member). We met after each batch of approximately five interviews to further refine the codebook, ensure coder agreement, and resolve any disagreements. Key themes were shared with the third author in June 2021 and December 2021 to finalize the main findings. The study received IRB approval from [the University of California, San Francisco].

## RESULTS

We interviewed twenty-one MA members who used their NEMT benefit at least once between 2019 and 2021. Sample characteristics are presented in Table 1 and included diversity by sex, self-identified race and ethnicity, urbanicity (defined by ZIP code), and age: 62% were female, 52% identified as White, 38% identified as Black or African American, 71% resided in urban areas, and 48% were over the age of 65.

**Table 1:** Demographic Characteristics of Study Participants

Demographic variables	Number of participants (*N* = 21)
Sex	
Male	8
Female	13
Age	
≤ 65	11
> 65	10
Race and ethnicity (self-reported)	
Black/African American	8
Other (Puerto Rican; Multiracial)	2
White	11
Population density	
Urban	16
Rural	5
Annual income ($) (self-reported)	
< 10,000	6
10,000–30,000	14
Declined to answer	1
Chronic conditions	
< 2	13
> 2	8
In-patient stay within last year	
Yes	15
No	6

Two major themes emerged during conversations with participants. First, participants considered NEMT essential, particularly because transportation services not only ensured access to medical appointments but also helped them mitigate social isolation, reduce financial insecurity, and manage their own medical needs. Second, the design and delivery of NEMT services presented logistical challenges for participants that took considerable time and energy to circumvent.

### Theme 1: Transportation as an Essential Service

Participants viewed transportation (including NEMT and other transportation options) as essential to supporting health care. Transportation was not a supplemental or alternative need but for the individuals we interviewed, described as a core component of accessing health care. As one individual poignantly stated: “How can you receive health care if you can’t get there?” This centrality of NEMT to access health services emerged in many interviews. For example, one participant who did not own a car specifically chose his insurance plan because of the associated transportation benefits. He lived in a metropolitan area and relied on NEMT to access all his medical appointments and laboratory visits. He shared that for him, “medical transportation is one of the most important services” and a key support for him to optimize his health while living with a chronic medical condition. Another participant recently moved to a more rural community and relied on NEMT to access primary care services. “Without having the transportation, I have nowhere to go. I mean, I miss my appointments and I can’t get the things I need from the store. It just won’t happen.”

#### Community Support and Isolation

Nearly half (48%) of the participants we interviewed lived alone and reported isolation. For these individuals, NEMT services were often the only means they used to leave their homes. One interviewee struggled with wound care and multiple chronic conditions. He only left his house for medical appointments. Another participant relied on a wheelchair for mobility and shared how the COVID-19 restrictions exacerbated her baseline isolation and largely left her homebound. “I basically don’t get around. I’ve been in the house since January of 2020 and the only places I’ve been it’s like to the hospital, which I use [the NEMT benefit] to get me to, or the doctor’s office … So, if I can’t get [NEMT] or somebody to do something for me or [I] can’t do it myself, I’m just stuck here because there’s no public transportation here at all for me.”

#### Navigating Medical Complexity

As expected, given the MA population we interviewed, participants managed substantial medical complexity: three quarters had an inpatient hospital stay in the previous year, approximately half had two or more chronic conditions, and half reported being disabled. Medical complexity contributed to an increased need for transportation to medical services (many participants navigated multiple weekly or monthly appointments for specialty and primary care) and decreased mobility. Several participants were no longer able to drive because of their medical conditions. For example, some had lost driver’s licenses because of poor vision, cardiac disease, or other health conditions that prevented them from safely operating a vehicle.

#### Intersection of Transportation and Financial Insecurity

Most participants we interviewed also were living with financial insecurity (see Table 1). This translated into little flexibility regarding alternative transportation services, such as using taxis or ride shares. For example, one woman relied on disability payments as her primary source of income. Since she lived in a community with no public transportation, NEMT benefits greatly reduced her monthly transportation expenses. “The benefit with the insurance is that they don’t charge us anything to go to a medical appointment, which is really very good because I’m on a fixed income.” Another participant shared that he needed to pay $40 for a taxi to get to his doctor’s appointments: “I just have social security [and] disability coming in…And if I tried to do that four or five times a month, that would destroy everything.”

#### Transportation Services as Empowerment

Participants thus relied on NEMT as an essential service to attend often frequent medical appointments. But they also shared how these services empowered them to manage their own medical needs and reduced their reliance on others. For example, one woman we interviewed had relocated to an urban area to live closer to her children. While her daughter helped by providing rides to some medical appointments and to the grocery store, the participant chose her MA plan specifically because the NEMT benefit gave her more independence. “I don’t want to rely, depend on my daughter. I thank God for her, but I’d rather do things on my own. That’s what the insurer is there for… That’s the whole purpose of me calling transportation so I can feel better of taking care of what I need to take care of.”

### Theme 2: Navigating the Complex Logistics and Unpredictability of Transportation

While individuals we interviewed appreciated—and often relied on—their NEMT benefits, they also experienced challenges using NEMT services. They described difficulty coordinating transportation vendors, timely and reliable pick up/drop off, and quality and predictability of route and vehicle type. Because participants viewed NEMT services as essential, they were invested in improving the design of services to ease use. To that end, they specifically suggested ways to increase service flexibility and reliability that could inform future NEMT policy and practice reform. In this section and Table 2, we highlight key challenges participants shared with us and potential solutions they suggested related to each of those challenges.

**Table 2:** Challenges to Using NEMT and Patient-Recommended Solutions

	Description	Supporting quotes	Patient recommended solutions
Scheduling rides	• Process takes too long • Requires too much advance notice	• Just give me an operator who can arrange my ride. **I have like 400 phone minutes a month. I really don’t want to waste them on hold** • I had to use it [NEMT benefit] to go to a doctor’s appointment about my [broken] shoulder, but **the first time I had to use [the county program] because I didn’t have five days to schedule with the [doctor]**	• Allow for scheduling via multiple modalities • Eliminate wait-times • Reduce 72-h window to 24-h window or allow for on-demand rides
Transport arrival	• NEMT may not arrive on time or at all	• Sometimes I call for a ride and **they never show up or, so I miss my appointments**, and I get charged for a copay • There was one time that they told me what the pickup time was, and **I went outside and sat in my driveway, waiting for them to come. And, they never came**	• Communicate arrival time and any changes through phone or text message • Enable vehicle tracking
Quality & predictability of ride	• Ride may not be accessible for member • Uncertainty in the type of ride makes planning hard for members	• The last couple times I tried to get on and **they said that they couldn’t accommodate me because they didn’t have anybody who could take a motorized wheelchair** • I was using the [NEMT] bus at one time, but sometimes they would pick you up on time and sometimes they wouldn’t. And I had to have a lift [i.e. a wheelchair ramp] to get on it. **And at some time they did not have a lift at the time, so I had to reschedule my appointment** • They **didn’t have anybody that can take me in a power chair**, I just gave up	• Communicate vehicle type and make • Ensure alignment between vehicle type deployed with patient mobility needs
After Appointment Pick-up	• Pick-up times and schedules were unpredictable • Members had long wait times for pick-up • Rides are sometimes shared, increasing transportation time	• They **left me sitting outside in the rain and never came and picked me up** • They’ll take you to where you going, and then they don’t come and pick you up at a proper time. I stayed there. **I was there for three hours after this place was closed, and they still didn’t pick me up so I caught the bus home** • One afternoon it was like I was riding all around town, and **it took four hours to get home, that what was a 20-min ride**	• Communicate pick up time through vehicle tracking apps for transparent expected arrival and ride duration

Emphasis added to patient described NEMT challenges

#### Coordinating Multiple Transportation Services

Participants often used multiple services to meet their transportation needs because of the limited scope of NEMT. For example, individuals relied on NEMT to get to their medical appointments but still needed transportation to access the grocery store or community events (e.g., senior center activities, social engagements with friends and family, and visiting the library). While people who had them appreciated that there were transportation programs that could help them meet both their medical and non-medical needs, they expended significant energy coordinating and navigating the eligibility rules and processes of multiple transportation programs. For example, one individual we spoke with was eligible for a state-based discounted transportation services. To reduce her out-of-pocket transportation costs, she would first use the 24 free rides provided by NEMT. Once these were used up, she would begin scheduling her state-covered rides, which cost her $2.75/ride. Another participant had access to a county program that provided $1 rides to any destination within a 15-mile radius from her house. She would use the county program for rides to nearby services (including medical visits) and save her NEMT rides for longer distances. Other individuals triaged their rides based on how much advance notice they had before their medical visit since NEMT required 72-hour notice for scheduling. One woman we interviewed who had a series of short-term appointments after a bone fracture shared with us, “I wish they had some kind of emergency transport, because I’m almost 75, and you can’t always schedule in advance the things that you need in a hurry.” In Table 3, we illustrate the decision-making process several individuals applied when navigating the complex transportation services landscape.

**Table 3:** Participants Engaged in Complex Decision-Making Processes to Choose Between Transportation Services

	Description of transportation needs and options	Quote	Factors considered when choosing transportation
1	Has more medical appointments than rides available on her plan Accesses county service when rides are less than 15 miles from home and NEMT for medical appointments farther than 15 miles	I use a courtesy transportation that’s ran by the X county for senior services and they use a dollar a ride, I’ll use Lyft or Uber, and it’s a dollar each way. It is limited to 15 miles, but it will take you…to a grocery store, to the recreation center, community center, anywhere I want to go within a 15-mile radius. So, that leaves out health appointments because my appointment, my doctors are located a little further out than 15 miles, so I’ve used [NEMT]	• Distance • Cost • Number of rides available
2	Has access to a senior bus program [PACE] that is a dollar a ride, a disability ride service, NEMT through insurance for medical appointments, and subsidized taxi vouchers through the county	I tend to just use PACE when I really need to get somewhere, or I use a cab quite frequently because through the senior citizen center here in X County, you’re able to buy $30 worth of taxi fares for $15. So, it ends up being a half price cab fare. So, I have something where I really need to get to in a hurry, that’s what I do	• Cost • Speed of travel
3	She uses her cancer center’s ride program for oncology appointment. She relies on caregiver to get to the grocery store and the NEMT ride for all other medical appointments	I got a caregiver and she takes me back and forth to the grocery store. To get to my appointment I have to call [NEMT]… I’m still seeing my cancer doctors and they call [Ride Share service] and they come pick me up	• Destination • Availability of resources
4	Navigates multiple services to access different destinations	With transportation, you would have to call three days ahead of time. And if I choose to get [county services], like I said, it has to be medical. I would weigh my options. Like, one day I called and set up transportation, and I called County Service, and I was trying to analyze to see who would get me there quicker and get me back home quicker	• Speed of travel • Scheduling time

#### Reliability, Predictability, and Quality of NEMT Services

Participants expressed concern around timely and reliable NEMT services. Multiple participants missed appointments because their ride was late or never arrived. One participant who used a wheelchair for mobility shared her frustration at missing appointments.

I went outside and sat in my driveway, waiting for them to come. And they never came… and this was an appointment that I had to go to my dialysis center. And so, I called. And first the guy hung up on me and then I called again and I said, “I’m sitting here waiting for you.” And he says, “Well, I was already there. I rang the doorbell and nobody was there.” And I said, “No, you didn’t.” I said, “I have been sitting in the driveway for the last forty-five minutes, nobody was here.” So, anyway, I ended up missing that appointment.

Participants expressed dismay around the lack of communication with schedule changes, especially given the advanced planning they were asked to provide when scheduling rides. As one participant expressed: “Just give somebody some notice, if you’re not going to be able to do something or if something gets cancelled… That’s kind of disheartening, too because a person can make other arrangements if they know. But if you don’t know, there’s nothing you can really do.”

The biggest source of frustration for participants was the lack of ride reliability following medical appointments. One participant who lacked alternative means of transportation shared, “I sit in the hallway [of the clinic] and I call them and when they come, they come. See, sometimes it has been a long time, but sometimes it’s not. You just have to wait until the cab come[s]… I usually get sick [and tired of] waiting like that, but I had to deal with it because that’s the only way I can get home.” Two participants stopped using NEMT after their ride never arrived to pick them up after an appointment. “I was done at the doctor, and ninety degrees out, and I was done at the doctor at 3:00. And the office is closed and everybody was leaving and it was 6:30 and I was still standing there.”

Participants also expressed concerns around the predictability and quality of route and vehicle. Participants recounted prolonged routes with multiple stops. “One afternoon it was like I was riding all around town, and it took four hours to get home, what was a 20-min ride.” Specifics of the ride (vehicle type, driver) were not typically disclosed to participants in advance, which created unpredictability and stress. One participant who used NEMT regularly shared that this was his biggest concern of NEMT. “The only thing that’s wrong in that process is that as a senior citizen and as a person who is not a car person, I never know what to look for. I’m standing there and I’m going like, ‘Well, someone’s going to come, but what am I looking for?’…. They use a combination of Lyft, cab drivers, and medical vans… So you never know what you’re going to get when you’re out there.”

#### Transportation Services for Individuals with Mobility Devices or Requiring Additional Support

Many participants relied on mobility devices or required additional assistance in navigating transportation services. Yet participants were sometimes assigned vehicles that did not match their mobility needs. For example, one woman who used a wheelchair stopped using NEMT because she never knew if the vehicle could take her wheelchair. “The last couple times I tried to get on and they said that they couldn’t accommodate me because they didn’t have anybody who could take a motorized wheelchair.” Other participants recounted that drivers refused to take their walkers or power chairs, or lacked necessary lifts, and as a result, they missed appointments. “I would make it clear, I was in a wheelchair and they would have to take my wheelchair…Then when the ride would get there…they didn’t want to take my wheelchair.”

## DISCUSSION

Transportation plays a foundational role in connecting individuals to housing, education, employment opportunities as well as medical care services. Because of the connective nature of transportation, it is also emerging as a high-priority area in many federal and state health care initiatives and research agendas.^15–17^ Yet little is known about patients’ experiences using existing health-care-related transportation services or about best practices for addressing patients’ transportation barriers. In this qualitative study, MA members described NEMT as an essential and valued service. Participants also highlighted the challenges associated with navigating NEMT services. Many existing transportation programs (including both NEMT and other community transportation services) require individuals to coordinate complex transportation logistics. Our findings underscore the importance of including patients’ perspectives in NEMT design and reveal opportunities to strengthen transportation benefits and implementation.

If transportation is so central to healthcare access, why is it so difficult to address? One of the answers to this question is that transportation access is influenced by both community resources and individual needs, each of which is deeply embedded in social and historical contexts. The types of transportation services available in any community are shaped by historical and structural racism, including exclusionary planning policies that have determined where public transportation lines are built and the opportunities for other ride services and active transportation.^18,19^ In addition, transportation access is contingent on an individual’s mobility, which affects access to traditional transportation services (e.g., buses, trains, and standard taxis). Yet NEMT benefits are largely designed as discrete supports to move individuals between points A and B (typically home and a medical appointment) and do not account for variability in patient resources and needs. The result is that each individual is independently responsible for navigating disjointed transportation environments.

NEMT offers a concrete opportunity to advance innovative transportation solutions. By elevating patients’ perspectives and experiences, our findings highlight concrete steps that can be applied to improve NEMT. First, NEMT services can be strengthened through more intentional coordination with other regional transportation services. Access to the grocery store, community events, family, and friends, are central to patient health and well-being.^11,20,21^ The fragmentation of current transportation services fails to sufficiently coordinate across sectors or leverage existing resources to advance this range of health-promoting behaviors. This contributes to additional work for patients and duplication of services. Second, payers and policymakers must work together to ensure the standardization of transportation quality to provide services that better align with patients’ needs, including the timely receipt of services and vehicles that meet the needs of patients with disabilities.

Our study findings should be interpreted in the context of several limitations. First, as a qualitative study, findings are not meant to be generalized to all NEMT users. We did, however, recruit a diverse sample by factors like a medical condition, sex, region, self-identified race, and urbanicity, and highlighted themes shared across interviews. Second, we recruited participants who used their NEMT at least once in 2019 and remained eligible for NEMT benefits through 2021, meaning patients needed to potentially recall a transportation event that occurred in 2019. In our sample, patients easily identified transportation services provided through their insurance and did not struggle to recall these events but we had no means to confirm rides. While our study was limited to a single payer, the NEMT broker in this study manages a third of the NEMT market in the US and contracts with multiple insurance providers (Medicaid and MA). Thus, our findings offer insight into service beyond our study population. Though social desirability bias is often a limitation in this type of research, in this study, we do not believe that it would have meaningfully influenced findings for two reasons. First, there was no clear direction for bias, said differently NEMT was not uniformly viewed as a positive or negative experience. Second, our participants shared many critiques of NEMT and suggestions for improvement. The eligibility period overlapped with the beginning of the COVID-19 pandemic, which impacted mode of care (in-person versus telehealth) and the availability of transportation services (e.g., reduced or closed public transit and changes in multi-rider vans.) This context likely influenced members’ experiences with NEMT.

Comprehensive approaches to transportation security will require re-envisioning transportation as a community resource rather than an individual one. NEMT is a vital service, especially given that the need for NEMT services is likely to grow as the US population ages. Indeed, NEMT spending is already estimated at $6 billion and is expected to hit $14 billion in 2024.^22^ In this study, some participants and their caregivers successfully navigated the complex transportation landscape, but many were left stranded. Ideally, NEMT vendors, payers, and health service providers will use our findings about patients’ experiences with NEMT to address long-standing gaps in transportation access and thereby improve health equity.
